# Nurses’ knowledge, attitude and practices on use of restraints at State Mental health care setting: An impact of in-service education programme

**DOI:** 10.17533/udea.iee.v39n1e12

**Published:** 2021-03-05

**Authors:** Sreevani Rentala, Sunanda Govinder Thimmajja, Prashant Bevoor, Raveesh Bevinahalli Nanjegowda

**Affiliations:** 1 Ph.D. in Nursing, Nursing professor. Dharwad Institute of Mental Health and Neurosciences, Dharwad, Karnataka, India. Email: sreevani.phd@gmail.com. Corresponding author National Institute Of Mental Health And Neurosciences Dharwad Institute of Mental Health and Neurosciences DharwadKarnataka India sreevani.phd@gmail.com; 2 Ph.D. in Nursing, Assistant Professor. Dharwad Institute of Mental Health and Neurosciences, Dharwad, Karnataka, India. Email: amogh_aniketh@yahoo.com National Institute Of Mental Health And Neurosciences Dharwad Institute of Mental Health and Neurosciences DharwadKarnataka India amogh_aniketh@yahoo.com; 3 RN.RM. Nursing officer. Dharwad Institute of Mental Health and Neurosciences, Dharwad, Karnataka, India. Email: prashantbevoor7@gmail.com National Institute Of Mental Health And Neurosciences Dharwad Institute of Mental Health and Neurosciences DharwadKarnataka India prashantbevoor7@gmail.com; 4 MD. Psychiatry. Professor and Head. Dept. of Psychiatry, Mysore Medical College, Mysore, IndiaEmail: raveesh6@yahoo.com University Of Mysore Mysore Medical College Mysore India raveesh6@yahoo.com

**Keywords:** restraint, physical, psychiatric nursing, health knowledge, attitudes, practice, restricción física, enfermería psiquiátrica, conocimientos, actitudes y práctica en salud, restrição física, enfermagem psiquiátrica, conhecimentos, atitudes e prática em saúde

## Abstract

**Objective.:**

To evaluate the effectiveness of short-term in-service education program in improving nurse’s knowledge, attitude and self-reported practices related to physical restraint use.

**Methods.:**

A quasi-experimental one group pre-post study was conducted involving nurses working at a tertiary mental health care setting, Dharwad, India. We provided 3 consecutive days of intensive restraint management education (total 6 hours-two hours per day) with a follow-up assessment after one month. The standard questionnaires on knowledge, attitude and practice regarding physical restraints were used as tools for measuring the impact of in-service education program. The program was conducted for a group of five to six nurses at a time. Teaching was done using lecture method, group discussion and demonstrations.

**Results.:**

Of the 52 nurses who participated in the study, 52% were male, 58.5% had a baccalaureate degree. The mean age of respondents was 33.3 years, the mean work experience was 6.7 years. The findings of the study revealed that the mean scores on the knowledge regarding physical restraints increased after the in-service education from 6.4 to 8.2 (*p*<0.001). The mean attitude scores improved from 18.5 to 23.1 (*p*<0.001). There was a significant difference in mean practice scores between pre and post-intervention phases (23.7 versus 25.4; *p*<0.001). There was a significant correlation between post-test knowledge, attitude and practice scores.

**Conclusion.:**

The in-service education program improved nurse’s knowledge, attitude and self-reported practice scores. This may lead to more effective restraints management by psychiatric nurses.

## Resumen

**Objetivo.:**

Evaluar la efectividad a corto plazo de un programa de educación en servicio para enfermeros, el cual tenía como fin mejorar los conocimientos, las actitudes y las prácticas auto-informadas en relación al uso de medidas de contención física.

**Métodos.:**

Se realizó un estudio cuasi-experimental con evaluación de pre y post-intervención de un grupo de enfermeros que trabajaban en un entorno de atención terciaria de salud mental en Dharwad (India). Durante tres días consecutivos, se hizo educación sobre el manejo de la contención física (un total de 6 horas, dos horas por día) con una evaluación de seguimiento después de un mes. Los cuestionarios estándar sobre conocimientos, actitudes y prácticas con respecto a la contención física se utilizaron como herramientas para medir el impacto del programa en servicio. Las sesiones se llevaron a cabo en grupos de cinco a seis enfermeras a la vez. La enseñanza se realizó mediante el método de conferencias, debates en grupo y demostraciones.

**Resultados.:**

De los 52 enfermeros que participaron en el estudio, el 52% eran hombres, el 58.5% tenía un título de bachillerato en enfermería (formación profesional de 4 años). La edad media de los encuestados fue de 33.3 años, con una experiencia laboral media de 6.7 años. Los hallazgos del estudio revelaron que las puntuaciones medias en el conocimiento sobre restricciones físicas aumentaron después de la educación en el servicio de 6.4 a 8.2 (*p*<0.001). Las puntuaciones medias de actitud mejoraron de 18.5 a 23.1 (*p*<0.001). También se observó una diferencia significativa en las puntuaciones medias de práctica entre las fases pre y post-intervención (23.7 versus 25.4). Hubo una correlación significativa entre los puntajes de conocimientos, actitudes y prácticas posterior a la prueba.

**Conclusión.:**

El programa de educación en el servicio mejoró el conocimiento, las actitudes y las prácticas auto-reportadas de los enfermeros, lo que puede conducir a un manejo más efectivo de la contención física por parte de los enfermeros psiquiátricos.

## Resumo

**Objetivo.:**

Avaliar a eficácia em curto prazo de um programa de educação em serviço para enfermeiros que buscam aprimorar conhecimentos, atitudes e práticas autorreferidas em relação ao uso de medidas de contenção física.

**Métodos.:**

Foi realizado um estudo quase experimental com avaliação pré e pós-intervenção de um grupo de enfermeiras trabalhando em um ambiente terciário de saúde mental em Dharwad (Índia). Educação sobre o manejo da contenção física (um total de 6 horas, duas horas por dia) foi dada por três dias consecutivos com uma avaliação de acompanhamento após um mês. Questionários padronizados sobre conhecimentos, atitudes e práticas em relação à contenção física foram usados ​​como ferramentas para medir o impacto do programa em serviço. As sessões foram realizadas em grupos de cinco a seis enfermeiras de cada vez. O ensino era feito por meio de palestras, discussões em grupo e demonstrações.

**Resultados.:**

Dos 52 enfermeiros que participaram do estudo, 52% eram homens, 58.5% tinham o título de bacharel em enfermagem (4 anos de formação profissional). A idade média dos entrevistados era de 33.3 anos, a experiência de trabalho média era de 6.7 anos. Se realizou o teste t pareado para encontrar a diferença média entre a educação pré e pós-formação em serviço. Os resultados do estudo revelaram que as pontuações médias no conhecimento sobre restrições físicas aumentaram após a educação em serviço de 6,4 para 8,2 (*p*<0.001). As pontuações médias de atitude melhoraram de 18.5 para 23.1 (*p*<0.001). Uma diferença significativa também foi observada nas pontuações médias de prática entre as fases pré e pós-intervenção (23.7 versus 25.4). Houve uma correlação significativa entre as pontuações de conhecimento, atitude e prática pós-teste.

**Conclusão.:**

O programa de educação em serviço melhorou os conhecimentos, atitudes e práticas autorreferidas dos enfermeiros, o que pode levar a um gerenciamento mais eficaz das restrições físicas pelos enfermeiros psiquiátricos.

## Introduction

Physical restraint is defined as the restriction of the patient’s movements and prevention of his/her moving freely by connecting physical or mechanical devices to the patient’s body or by means of a short term physical force applied by the healthcare personnel.(1) These are widely practiced in psychiatric care settings and at times this would be the last option available to reduce treatment interference and keep the patient safe.(2) Studies show that in many countries,>20% of psychiatric patients are restrained physically at some point during their hospitalization.(3) A study conducted in the Indian setting reported that restraint was used as a method of control for violent, suicidal, agitated and delirious patients.(4) In nursing home settings the prevalence rates of restraints use ranged from 19 to 84.6%, while it is reported at 34% in rehabilitation settings.(5) Previous study showed the relatively high prevalence rate of restraint use in local nursing homes and long-term care facilities, clinical observations by many health professionals endorse the widespread use of physical restraints.(6)

Although the restraint intervention is inherently designed to protect patients from harm to self or others, it is associated with many potential complications. Many studies have shown the negative effects of physical restraint on both patient and healthcare personnel.(7) A survey of 142 patients identified the frequency of potentially harmful events and associated psychological distress. This procedure further stimulates aggression among patients and damages the therapeutic relationship between the healthcare personnel and the patient.(8) It is also contrary to the treatment principles and patient dignity.(3) To ensure good quality care the teaching restraint use for nurses deserves better attention.(6) It is the nurse’s professional responsibility to ensure the safety of the individual in the hospital environment. Therefore, nurses must know the possible complications of physical restraint and follow up patients who are physically restrained. Psychiatric nurses are responsible for establishing a safe and therapeutic environment for patients, maintaining it and ensuring optimal clinical restraint surveillance based on the restraint application stan dards. (9) In this context, psychiatric nurses should have adequate knowledge and skill in application of physical restraints.

Several studies have demonstrated that the knowledge of nurses regarding proper use of physical restraints is not satisfactory. (10) Furthermore, some studies showed that nurses have mixed-feelings about the use of physical restraints.(6) A study conducted in Turkey reported that a low percentage of nurses knew the complications of physical restraints.(11) A similar study conducted in Hong Kong determined the inadequate knowledge of nurses about physical restraint. They exhib ited negative attitude towards restraint application.(12) In another study moderate knowledge and attitude with strong intension to use physical restraint was found among nurses.(13) Restraining is a highly preferred practice in psychiatric wards and use of alternative procedures before restraining the patient is minimal.(14) Less than half of the nurses considered alternatives to physical restraint, while most of them did not understand the reasons for using them. A study among psychiatric nurses showed ambivalent attitude towards use of physical restraints among mental health consumers.(15) Another study highlighted some important misunderstandings among nurses regarding use of physical restraints.(16) Further, it was argued that views and attitudes of nurses’ towards the use of physical restraints may create a conflict with patients’ rights and their autonomy in taking decisions.(17) A recent study conducted in Indian context indicated moderate knowledge and poor attitude among nurses regarding restraint use. This study recommended development of nursing guidelines and training of nursing personnel for proper use of physical restraints.(18)

The knowledge, attitudes and intentions of nurses towards physical restraint use are essential factors that may contribute to effective physical restraint practice.(13) Finding from earlier studies serve as a supporting reason for recognizing the importance of educating nurses on physical restraints. The best approach to improve knowledge and attitudes towards the use of physical restraint is through educational interventions.(12) Providing accurate knowledge, imparting proper skills, cultivating positive attitude, and rectifying irregularities in physical restraint use are all necessary for nurses to improve patient care.(17)

There are some research studies that demonstrate the effectiveness of education interventions on the knowledge, attitude, and practice of nurses towards physical restraint and the frequency of physical restraint use in hospitals.(19, 20) In these studies the duration of education programs varied from 1 hour to 12 weeks.(20) A number of previous studies measured the knowledge, attitude and practices of nursing staff towards the use of restraints in acute, elderly and psychiatric care settings. However, not many studies examined the effectiveness of education program on improving knowledge attitude and practice skills among nurses on physical restraints. Further, there were no formal studies on this issue from India.(21) Hence, the present study was aimed to determine the effectiveness of short-term in-service education program in improving nurse’s knowledge, attitude and self-reported practices related to physical restraint use among nurses working in mental health care setting.

## Methods

Research design and settings. A quasi-experimental study with one group pre-post test design was carried out at a tertiary mental health care setting in Karnataka, India. It is a state government mental health care setting with 212 beds. Clinical services comprise of inpatient, out-patient, emergency and rehabilitative services. Both voluntary and involuntary admissions along with forensic cases are catered to.

Sampling and participants. The sample consists of 52 registered nurses working at mental health care setting, Dharwad, India. Convenience sampling technique was applied. Of the 59 staff nurses working in the mental health care setting while 3 were on long leave and4 refused to participate, the remaining 52 gave their consent to participate. The data was collected between August 2017andOctober 2017. Inclusion criteria were: (a) registered nurses (b) with minimum 6 months experience in psychiatric wards (c) willing to participate.

Instruments. Demographic information includes gender, age, education qualification and total years of experience in nursing. The standard questionnaire on knowledge, attitude and practice regarding physical restraints was used to collect data from participants. This scale was developed by Janelli et al 1994(22) in the USA for nursing homes. This scale was selected as it was previously used in India and demonstrated acceptable levels of validity and reliability. The questionnaire consists of 37 items divided into three parts. Part 1with 11 items deals with the nurse’s level of knowledge towards the use of restraints. Each correct answer was scored as 1 and incorrect as 0 with the total possible score ranging from 0 to 11. Higher score denotes higher knowledge about physical restraint use. Part 2 with 14 items measures nursing practices. Participants were asked to respond on a 3-point Likert scale about whether they always, sometimes or never performed these practices. Each item was given a score of 2 for always, 1 for sometimes, 0 for never (potential range 0-28). Reverse scoring was done for negative items. The respondent’s score correlated positively with his or her level of proficiency at using physical restraints properly. Part 3 with 12 items measure the attitudes of nurses toward the use of restraints. The participants were asked to respond on a 3-point Likert Scale about whether they strongly agree, agree, disagree or strongly disagree. Each item was given a score of 3 for strongly agree to 0 for strongly disagree. Higher scores thus reflected positive attitude while lower scores reflected negative attitude (potential range 0-36). Reverse scoring was done for negative items. The test-retest reliability coefficients for individual sections (section 1, 2 and 3) of the questionnaire were examined by administering the same instrument repeatedly to 15 nursing students at a 2-week interval. The reliability coefficients for the knowledge, attitudes and practice scales used in this study were 0.75, 0.81 and 0.94 respectively. Content validity for the intervention program was established by taking opinion from 7 experts.

Data collection. Tools were administered to participants on day one. After the pre-assessment participants were attended three consecutive days in-service educations. Post-test was conducted 1-month after the in-service education.

Intervention (In-service education). Participants were invited to the in-service education program for three consecutive days (total 6hrs - two hours per day). The program was conducted for a group of five to six nurses at a time. Total 10 groups completed in-service education.

A structured teaching plan for in-service education was developed in line with the institution policy, expert panel’s opinions, and literature review regarding minimising physical restraints use in hospitals. The intervention focused on the myths and facts relating to physical restraints use, physical restraint alternatives, and ethical issues, use of de-escalating methods, handling psycho-social issues, proper application and imparting care during restraints use especially for patients with mental disorders. Teaching was done using lecture method, group discussion and demonstrations. Video teaching and case scenarios were used for group discussion. Demonstration mainly focused on application of physical restraints and safety precautions. A panel of 5 psychiatric nursing experts and psychiatrists verified and validated content of the educational intervention.

Data analysis. The data were analysed using the Statistical Package for Social Science version 22. Descriptive statistics were used to describe demographic variables. A paired t-test was used to compare pre-mean and post-mean knowledge, attitude and practice scores. Cronbach’s alpha was used to establish reliability of the instruments. Pearson’s correlation coefficient was used to correlate post-test knowledge, attitude and practice scores on physical restraints among nurses.

Ethical considerations. Ethical approval was obtained from the institutional ethics committee before conducting the study. Participation was voluntary and written informed consent was obtained from the participants. The study protocol was approved by the Institute's Ethics Committee.

## Results

Nurses demographic and professional characteristics. A total of 25 (48%) female and 27 (52%) male nurses participated in this study, mean age being 33.29 years (SD=7.39). Nearly half of the participants (58.5%) were graduate nurses and the another 40.5% were diploma nurses. Mean work experience for participants was 6.71(SD=6.80).

Effect of training program on nurses’ knowledge attitude and practice regarding physical restraints. A paired sample t-test demonstrated significant improvement in nurses’ knowledge, attitude and self-report practice between pre and post-test scores. There was a significant increase in the mean knowledge scores, which increased from a mean of 6.42 (SD=1.56) in the pre intervention to a mean of 8.20 (SD=1.44) in the post intervention phase (t=-6.48, *p*<0.001). Mean attitude scores improved during the pre-intervention (mean=18.50, SD=3.48) to post intervention period (mean=23.12, SD=4.91) (t=-3.77, *p*<0.001). There was a significant difference in mean practice scores between pre intervention (mean=23.67, SD=2.41) and post intervention phase (mean=25.44, SD=2.21) (t=-5.72, *p*<0.001) (Table 1).

Effect of training program on nurses’ knowledge attitude and practice regarding physical restraints. A paired sample t-test demonstrated significant improvement in nurses’ knowledge, attitude and self-report practice between pre and post-test scores. (Table 1).

**Table 1:** Comparison of pre-test and post-test knowledge, practice and attitude scores regarding physical restraints use among 52 nurses

Parameter	Max. Score	Pre-test Mean (SD)	Post-test Mean (SD)	***t*-test**	***p*-value**
Knowledge towards use of restrains	11	6.42 (1.56)	8.20 (1.44)	-6.48	<0.001
Nursing practices towards use of restraints	28	23.67(2.41)	25.44 (2.21)	-3.77	<0.001
Attitude regarding use of restraints	36	18.50(3.48)	23.12 (4.91)	-5.72	<0.001

Correlation between post-test knowledge, practice and attitude scores regarding physical restraints among nurses. Pearson’s correlation coefficient test demonstrated significant positive correlation between post-test knowledge, practice and attitude scores on physical restraints among nurses (Table 2).

**Table 2:** Correlation between post-test knowledge, practice and attitude scores regarding physical restraints among 52 nurses

Variables	Knowledge	Practice	Attitude
Knowledge	1	0.290*	0.333**
Practice	0.290*	1	0.267*
Attitude	0.333*	0.267*	1

*p<0.05, **p<0.01

## Discussion

Physical restraint is commonly used as a measure of protection for psychiatric patients. Long-term use of physical restraints can lead to multiple medical, psychological and functional problems. Thus, the nurses need to be educated and updated to anticipate and recognize risky problems like abrasion at restraint site, incontinence of urine and stool, dehydration and decrease in functional status. Results show that the 3 days in-service education program improved nurse’s knowledge, attitude and self-reported practices on physical restraint use. Some studies have reported similar findings.(13)

This study reported significant improvement in knowledge scores among nurses post in-service educational program. The nurses participated in group discussion and lecture sessions which enabled them to differentiate between myths and facts of physical restraints. Present study results are in line with previous study results which showed a significant increase in mean knowledge, attitude and practice scores and a significant decrease in the mean intention scores of nurses in use physical restraint after educational intervention.(23) It is recommended that in-service training program should cover misconceptions regarding physical restraint use, ethical issues and how to cope with feelings while using physical restraints.

The mean attitude scores of 18.50 at pre-intervention level improved to 23.12, after attending the in-service educational program and this improvement was statistically significant. In the present study case scenarios were used for group discussions to clarify participants’ perceptions. Relevant education programs may need to include more problem-based case scenarios and discussions related to ethical issues to clarify nurse’s perceptions.(24) Scores on self-reported practice of physical restraint use improved after intensive in-service educational program. Application of physical restraints was demonstrated to improve practice skills. One study emphasized that nurses recognized a need for continuing education on restraint to improve their practices.(24) In another study nurses who had received on-the-job training performed better than those who had received no training related to knowledge and practices regarding physical restraint use.(25) This educational program may assist nurses to consider alternative measures before using physical restraints.

In the present study, significant positive correlation was found between post-test knowledge, practice and attitude scores on physical restraints among nurses. This shows that knowledge, attitude and practice are interrelated. With an improvement in level of knowledge attitude and practice also improved. Similar findings were reported by previous studies,(25) wherein a significant positive correlation was found between nurse’s practice score, knowledge and attitude scores. Similarly, in Eskandari *et al.* 2017(13) study a positive correlation was found between knowledge, attitude and practice of nurses towards application of physical restraints on patients.

Educational programs are easier ways to improve nurses practice skills. The care settings and government should support educational programs and impart knowledge and skills regarding use of physical restraints. Hospital administrators should plan in-service education for all nurses working in various wards of tertiary care hospitals. If physical restraint is to be practiced, staff nurses must not only understand their proper use but also their negative consequences.(25)

The present study has few limitations. Data were collected from only one hospital of Karnataka, India limiting the external validity of the results. Nurse’s practices regarding the use of physical restraint were assessed by a self-administered questionnaire which might not reflect actual behavior of nurses. This study may contribute to filling the gaps in nursing knowledge, improve skills and practice knowledge in physical restraint use in psychiatric hospitals. It may also assist the nurses in creating a supportive environment for use of alternative methods so as to reduce the use of physical restraints. 

The conclusion of this study is that results showed a significant increase in the mean knowledge, attitude and self-reported practice scores among nurses in use of physical restraints after their participation in the in-service educational program. Findings highlight the need to provide a short-term in-service education program on physical restraint use in mental health care settings. Study recommends the regular participation of nursing staff and other health care personnel in in-service education programs with a focus on ensuring patient safety, consequences of restraint use, alternative methods to restraints, care of patient with restraints, ethical and legal implications involved in restraining procedure.
